# Electroencephalographic differences between waking and sleeping periods in patients with prolonged disorders of consciousness at different levels of consciousness

**DOI:** 10.3389/fnhum.2025.1521355

**Published:** 2025-02-17

**Authors:** Keke Li, Man Li, Wanqing Liu, Yanzhi Wu, Fang Li, Jingwei Xie, Shaolong Zhou, Sen Wang, Yongkun Guo, Jiahui Pan, Xinjun Wang

**Affiliations:** ^1^Department of Neurosurgery, The Fifth Affiliated Hospital of Zhengzhou University, Zhengzhou, China; ^2^School of Automation Science and Engineering, South China University of Technology, Guangzhou, China; ^3^Department of Neurosurgery, The Third Affiliated Hospital of Zhengzhou University, Zhengzhou, China; ^4^Henan Engineering Research Center for Prevention and Treatment of Brain Injuries, Zhengzhou, China; ^5^Henan Key Laboratory of Brain Science and Brain Computer Interface Technology, Zhengzhou, China; ^6^School of Software, South China Normal University, Nanhai Software Technology Park, Foshan, Guangdong Province, China

**Keywords:** polysomnography, prolonged disorders of consciousness, CRS-R, fractal dimension, Teager–Kaiser energy operator

## Abstract

**Objective:**

This study aimed to explore differences in sleep electroencephalogram (EEG) patterns in individuals with prolonged disorders of consciousness, utilizing polysomnography (PSG) to assist in distinguishing between the vegetative state (VS)/unresponsive wakefulness syndrome (UWS) and the minimally conscious state (MCS), thereby reducing misdiagnosis rates and enhancing the quality of medical treatment.

**Methods:**

A total of 40 patients with prolonged disorders of consciousness (pDOC; 27 patients in the VS/UWS and 13 in the MCS) underwent polysomnography. We analyzed differential EEG indices between VS/UWS and MCS groups and performed correlation analyses between these indices and the Coma Recovery Scale-Revised (CRS-R) scores. The diagnostic accuracy of the differential indices was evaluated using receiver operating characteristic (ROC) curves.

**Results:**

1. The fractal dimension (Higuchi’s fractal dimension (HFD)) of patients in the MCS tended to be higher than that of patients in the VS/UWS across all phases, with a significant difference only in the waking phase (*p* < 0.05). The HFD in the waking phase was positively correlated with the CRS-R score and exhibited the highest diagnostic accuracy at 88.3%. The Teager–Kaiser energy operator (TKEO) also showed higher levels in patients in the MCS compared to those in the VS/UWS, significantly so in the NREM2 phase (*p* < 0.05), with a positive correlation with the CRS-R score and diagnostic accuracy of 75.2%. The *δ*-band power spectral density [PSD(δ)] in the patients in the MCS was lower than that in those in the VS/UWS, significantly so in the waking phase (*p* < 0.05), and it was negatively correlated with the CRS-R score, with diagnostic accuracy of 71.5%.

**Conclusion:**

Polysomnography for the VS/UWS and MCS revealed significant differences, aiding in distinguishing between the two patient categories and reducing misdiagnosis rates. Notably, the HFD and PSD(*δ*) showed significantly better performance during wakefulness compared to sleep, while the TKEO was more prominent in the NREM2 stage. Notably, the HFD exhibited a robust correlation with the CRS-R scores, the highest diagnostic accuracy, and immense promise in the clinical diagnosis of prolonged disorders of consciousness.

## 1 Introduction

Prolonged disorders of consciousness (pDOC) are defined as pathological states characterized by a loss of consciousness that lasts for more than 28 days following brain injury from various causes (Cheapen et al., 2017). This category includes the vegetative state (VS)/unresponsive wakefulness syndrome (UWS), in which patients appear awake but show no behavioral signs of consciousness, and the minimally conscious state (MCS), in which patients demonstrate fluctuating awareness and can respond appropriately to certain stimuli (Septien and Rubin, 2018). As critical care medicine advances, an increasing number of patients with acute brain injuries survive, leading to a rise in pDOC cases. This trend poses significant burdens on families and society (Kang et al., 2022). One of the challenges in clinically diagnosing and treating these patients is accurately determining their level of consciousness and distinguishing between the MCS and VS/UWS (Calabrò et al., 2016). This distinction has important ethical and therapeutic implications as patients in the MCS may experience pain or suffering and could benefit from analgesic treatments or other interventions aimed at improving their quality of life. In addition, the prognosis for MCS is generally more favorable than that for VS/UWS, making it crucial to encourage families to adopt a proactive approach in treating their loved ones (Thibaut et al., 2019). However, the differential diagnosis between the MCS and VS/UWS is extremely challenging and is associated with a high rate of misdiagnosis. Currently, the Coma Recovery Scale-Revised (CRS-R) is regarded as the gold standard for diagnosis, yet its misdiagnosis rate can be as high as 43%, due to both subjective factors (variability in scores among different raters) and objective factors (such as patients’ motor deficits and significant fluctuations in levels of consciousness; Giacino et al., 2004). For example, a study by Bodien et al. found that some patients diagnosed with VS/UWS using the CRS-R exhibited detectable abilities to perform cognitive tasks, as shown by functional magnetic resonance imaging (fMRI) and electroencephalogram (EEG), suggesting a certain level of consciousness that is often misdiagnosed in the clinic in this population (Bodien et al., 2024). There is an urgent need for new diagnostic methods to aid in accurate diagnosis.

Numerous studies have investigated the role of various diagnostic tools, such as EEG, fMRI (Bodien et al., 2024), positron emission tomography–computed tomography (PET-CT; Stender et al., 2014), and polysomnography (PSG), in diagnosing pDOC (Kondziella et al., 2020; Stender et al., 2014). Among these, EEG has been the focus of extensive research, revealing two primary types of indicators for differentiating between the consciousness states: linear and non-linear metrics. The linear indicators include the spectral power of different EEG bands, power ratios between frequency bands, and variations between closed and open eye states (e.g., alpha blocking; Schmidt et al., 2013). In contrast, the non-linear metrics encompass fractal dimensionality, entropy, and EEG microstates (Porcaro et al., 2022). Notably, Porcaro et al. found that the likelihood of distinguishing between the MCS and VS is greater when using Higuchi’s fractal dimension (HFD) compared to traditional linear methods (Porcaro et al., 2022). On the other hand, PET-CT and fMRI are less frequently utilized in clinical practice due to challenges such as high costs and difficulties in patient cooperation.

In contrast, PSG offers distinct advantages. Unlike neuroimaging techniques such as fMRI and PET-CT, PSG provides a direct measurement of neuronal activity in the brain. In addition, compared to short-term EEG, PSG is more sensitive to changes in brain network dynamics throughout the patient’s wake–sleep cycle. Duclos et al. found that improvements in consciousness and cognitive function were closely associated with enhancements in sleep–wake quality, highlighting a significant relationship between the sleep brain network and the conscious brain network (Duclos et al., 2017). Therefore, investigating polysomnographic differences in patients with pDOC is crucial for assessing their levels of consciousness. HFD is a time-series-based non-linear metric related to brain activity and brain network complexity (Walker et al., 2022). The Teager–Kaiser energy operator (TKEO) is a non-linear metric related to signal energy that quantifies instantaneous changes in EEG signal energy, emphasizing changes in the frequency and amplitude of the signal, thus responding to the strength of brain function (Shi et al., 2022). The *δ*-band power spectral density [PSD(δ)] is a commonly used linear metric, with larger values generally representing poorer awareness (Stefan et al., 2018). In this study, we analyzed PSG data from 40 patients with pDOC (27 in the VS/UWS and 13 in the MCS) to explore three discrepancies between the VS/UWS and MCS, calculating the diagnostic efficacy of these discrepancies separately.

## 2 Methods

### 2.1 Data collection and clinical evaluation

This study was a prospective cohort investigation involving 60 patients with pDOC who underwent PSG testing at the Fifth Affiliated Hospital of Zhengzhou University from 1 January 2024 to 31 July 2024. Basic clinical information was collected, including age, gender, etiology, and the duration of DOC. The inclusion criteria were as follows: 1. Age between 18 and 80 years; 2. DOC duration of 28 days or more; 3. no history of seizures and no use of antiepileptic, sedative, or other neuroexcitation-inhibiting medications before and after enrollment; 4. stable clinical status; 5. diagnosis of the VS/UWS or MCS based on the CRS-R scale; and 6. voluntary participation from the patient’s legal representative after reviewing the informed consent form. The exclusion criteria included: 1. History of psychiatric or neurodegenerative diseases prior to the onset of the condition; 2. severe cardiac, hepatic, or renal dysfunction or other significant comorbidities; and 3. the presence of a large cranial defect that would interfere with electrode-scalp contact. The enrolled patients were evaluated by two trained neurosurgeons who administered the CRS-R for five consecutive days. Based on the highest scores obtained, the patients were classified into either the VS/UWS group or the MCS group. In cases of discrepancies in the final scores, the supervising physician was consulted to reassess the patients and determine the final scoring results. Subsequently, a professional neurophysiologist conducted PSG tests on the patients. The study was conducted in accordance with the ethical standards of the Declaration of Helsinki and received approval from the Ethics Committee of the Fifth Affiliated Hospital of Zhengzhou University (Ethics No. KY2024006-K02). Before enrollment, the legal representatives of the patients were informed about the study, and written consent was obtained, with the option to withdraw from participation at any time.

### 2.2 PSG testing

Data were recorded using a mobile polysomnograph (NicoletOne, United States) with a sampling rate of 256 Hz, a high-pass filter set at 0.5 Hz, and a low-pass filter set at 40 Hz. The setup included 16-lead EEG, two-channel electrooculography, and three-channel electromyography. All recordings were conducted in the patient’s ward, and it was communicated in advance to the supervising physician and nursing staff that nursing interventions should be minimized during the testing. The experimenter checked electrode impedance after each intervention and replaced the electrodes when the impedance exceeded 5 kΩ. The patients remained in stable condition for at least 1 week before the recording, and their individual medication regimens were kept consistent. The recordings commenced at approximately 4:00 PM and concluded at 8:00 AM the following day.

### 2.3 Sleep score

The AASM Sleep and Associated Events Scoring Manual is the most widely used guideline for sleep staging (Morgenthaler et al., 2006). In this study, we adopted the sleep-scoring method proposed by Rossi et al. (2018), which aligns closely with the AASM guidelines while incorporating some modifications to account for the unique sleep patterns observed in the patients with disorders of consciousness (Cologan et al., 2013).

The two raters first reached a consensus on the sleep-scoring criteria before independently scoring each segment of the sleep EEG. Any discrepancies in period staging and arousal identification were subsequently reviewed and resolved. Uncertain periods were excluded from statistical analysis to maintain data integrity.

### 2.4 EEG data analysis

#### 2.4.1 Data preprocessing

The EEG signals were re-referenced using an average reference montage. The signals were filtered with a 50 Hz notch filter and a bandpass filter set between 0.5 and 40 Hz. To ensure consistency across the channels, each signal was normalized. For improved computational efficiency, the signals were downsampled to 100 Hz, and EEG was segmented into consecutive 30-s cycles. The time periods in which the voltage range of the EEG channel exceeded 100 μV were excluded from further analysis. Subsequently, the EEG traces were carefully reviewed, and any segments contaminated with residual artifacts were discarded. A text file containing sleep stage vectors was then loaded at a sampling frequency of 1/30 and upsampled to match the sampling frequency and duration of the EEG signal. Finally, we categorized the entire EEG data, after artifact removal, into wakefulness, NREM1, NREM2, NREM3, and REM phases according to the sleep-scoring method proposed by Rossi Sebastiano et al.

#### 2.4.2 Feature extraction

HFD is a method used to estimate the dimensionality of discrete time series data, serving as a quantitative measure of the inherent complexity or similarity of a signal or geometric structure (Walker et al., 2022). This method has been widely applied in sleep studies as it allows for the direct estimation of the fractal dimension from time series data without requiring prior transformations or embedding. The standard approach of HFD consists of the following steps: (1). For each sample i of an EEG segment Sj, calculate the absolute difference between Sj (i) and Sj (i + k) values (i.e., the samples at distance k), considering k = 1, klin. (2). These absolute differences are multiplied by a normalization factor that considers the sample size of the number of available samples for each k-value. (3). For each k, L(k) is calculated by summing the resulting values for all EEG segment samples and dividing by k. (4). By definition, if the value of L(k) is proportional to k-D, then the curve is a fractal curve of dimension D. If L(k) is proportional to k-D, klin, then log(k) and log(L(k)) have a linear relationship. In particular, from the log(L(k)) vs. log(k) curve (hereafter referred to as k), the linear coefficient of D as a regression line can be estimated by ordinary least squares. (5). Choose klin as the largest k for which L(k) is proportional to k-D. The fractal dimension D computed using the Higuchi method is in the linear region of the k-curve, i.e., k ≤ klin, while the non-linear region of the k-curve, i.e., k > klin, is usually not considered.

The TKEO is a non-linear energy tracking operator that measures the energy and frequency content of a signal, effectively determining the instantaneous energy of non-smooth signals. The discrete wavelet transform (DWT) has been successfully applied to EEG detection. Since the smoothness of the Daubechies 4 (db4) wavelet makes it more suitable for detecting changes in EEG signals, the DWT with the function db4 wavelet was used in this study. The DWT provides near-optimal time-frequency localization that decomposes EEG signals into seven different bands, corresponding to various brain rhythms. The signals were categorized into delta (A7), theta (D7), alpha (D6), beta (D5), gamma (D4), high gamma (D3), ripple (D2), and fast ripple (D1). Based on the TKEO method and the DWT, a combined global TKEO-DWT detection method is proposed, which analyzes the signal in both time and frequency domains.

The PSD quantifies the power distribution of a signal across various frequency components (Stefan et al., 2018). *δ*-wave brainwave frequencies typically range from 0.5 to 4 Hz and are closely associated with deep sleep stages. In this study, the Welch method was employed to compute the PSD for each time segment.

For detailed calculations of the characteristics mentioned above, please refer to Annex 1.

### 2.5 Statistical analysis

Continuous variables were reported as mean ± standard deviation, while non-normally distributed variables were expressed as quartiles. Categorical variables were presented as counts and/or frequencies. The Shapiro–Wilk normality test was conducted for the continuous variables. One-way ANOVA was utilized to identify indicators of variability between the MCS and the VS groups. The correlation between the variability indicators and the CRS-R scores was assessed using Spearman’s correlation analysis. In addition, the diagnostic accuracy of the variability indicators was evaluated using receiver operating characteristic (ROC) curves.

## 3 Results

### 3.1 Demographic results

**Figure 1:**
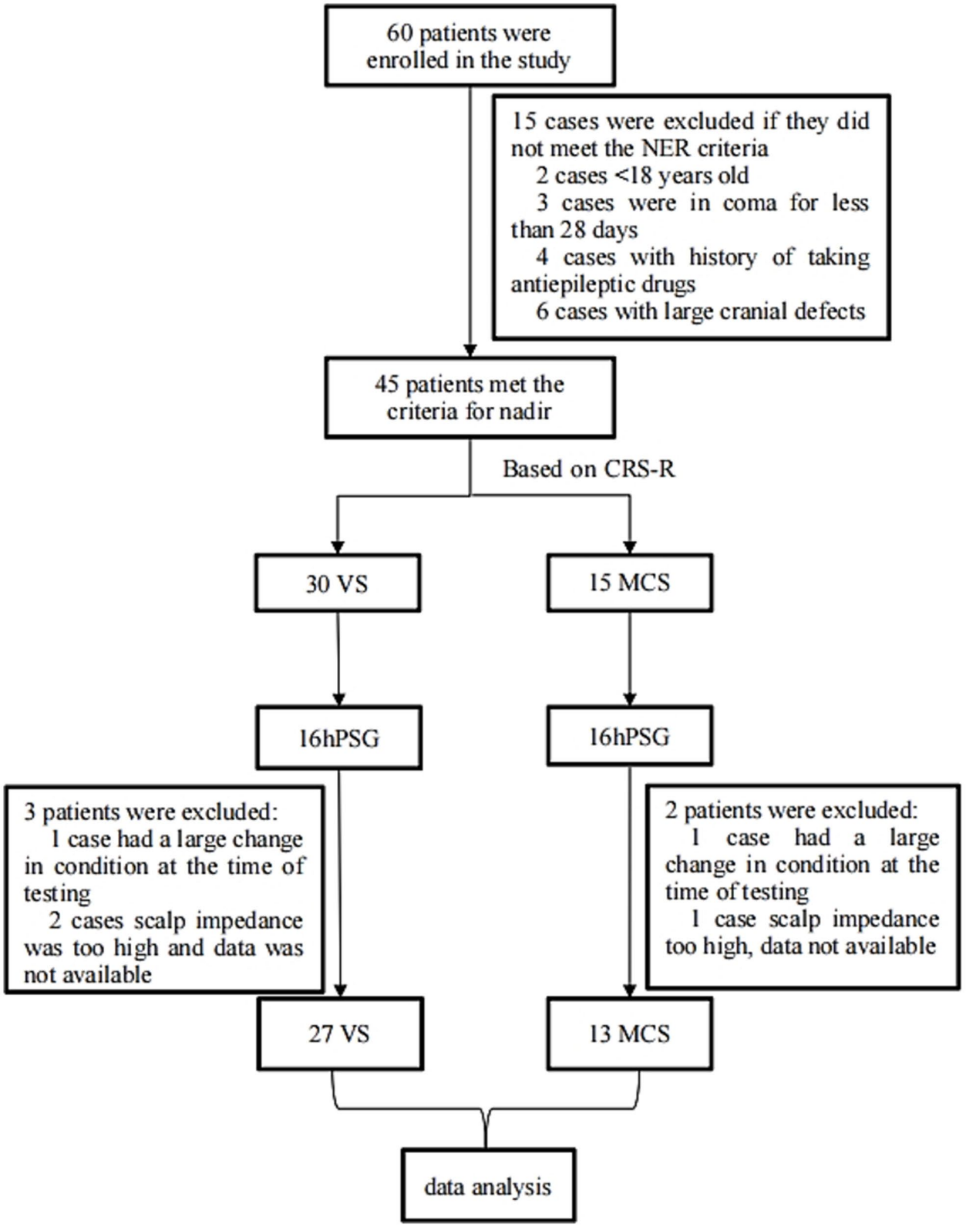
Flowchart of the study. CRS-R, Coma Recovery Scale-Revised; PSG, polysomnography.

A total of 60 patients with pDOC were initially included in the study. The study excluded 15 patients for failing to meet the inclusion criteria. Ultimately, 27 patients in the VS/UWS and 13 in the MCS were included in the analysis. The research subject and flowchart are shown in Figure 1. There were no significant differences in age, gender, the duration of DOC, or etiology between the two groups (*p* > 0.05). Basic clinical information is presented in Table 1.

**Table 1:** Basic clinical characteristics and sleep EEG characteristics of the patients.

	Total	MCS	VS/UWS	*p*-value
Patients (n %)	40	13 (32.5)	27 (67.5)	–
Sex (male n %)	19	8 (61.5)	11 (40.7)	0.217
Age (years)	54 ± 12.93	55.46 ± 7.03	53.3 ± 15.05	0.539
Etiology (traumatic brain injury n %)	12	4 (30.8)	8 (29.6)	0.941
Duration of coma (days)	79.78 ± 72.18	56.92 ± 42.67	90.78 ± 81.17	0.168
CRS-R	6.5 (5–8)	10 (8–11.5)	5 (5–7)	<0.001*
HFD				
Wake	1.47 ± 0.13	1.59 ± 0.10	1.42 ± 0.10	<0.001*
NREM1	1.43 ± 0.10	1.46 ± 0.12	1.42 ± 0.08	0.206
NREM2	1.41 ± 0.09	1.43 ± 0.08	1.40 ± 0.09	0.410
NREM3	1.13 ± 0.49	1.22 ± 0.38	1.08 ± 0.53	0.400
REM	1.34 ± 0.40	1.43 ± 0.11	1.30 ± 0.48	0.317
TKEO				
Wake	1.98 ± 0.93	2.25 ± 0.84	1.85 ± 0.96	0.214
NREM1	1.74 ± 0.7	2.07 ± 0.80	1.58 ± 0.71	0.054
NREM2	1.97 ± 1.12	2.62 ± 1.20	1.65 ± 0.94	0.009*
NREM3	2.14 ± 1.52	2.31 ± 1.20	2.07 ± 1.66	0.646
REM	1.51 ± 0.83	1.81 ± 0.90	1.37 ± 0.77	0.113
PSD(δ)				
Wake	0.43 ± 0.07	0.40 ± 0.06	0.45 ± 0.07	0.017*
NREM1	0.43 ± 0.08	0.41 ± 0.05	0.44 ± 0.09	0.237
NREM2	0.46 ± 0.08	0.43 ± 0.06	0.47 ± 0.09	0.093
NREM3	0.39 ± 0.20	0.33 ± 0.20	0.41 ± 0.20	0.241
REM	0.38 ± 0.15	0.37 ± 0.12	0.39 ± 0.16	0.701

CRS-R, Coma Recovery Scale-Revised; HFD, Higuchi’s fractal dimension; TKEO, Teager–Kaiser energy operator; PSD(*δ*), δ-band power spectral density; *indicates statistically significant differences.

### 3.2 Results of the PSG analysis

**Figure 2:**
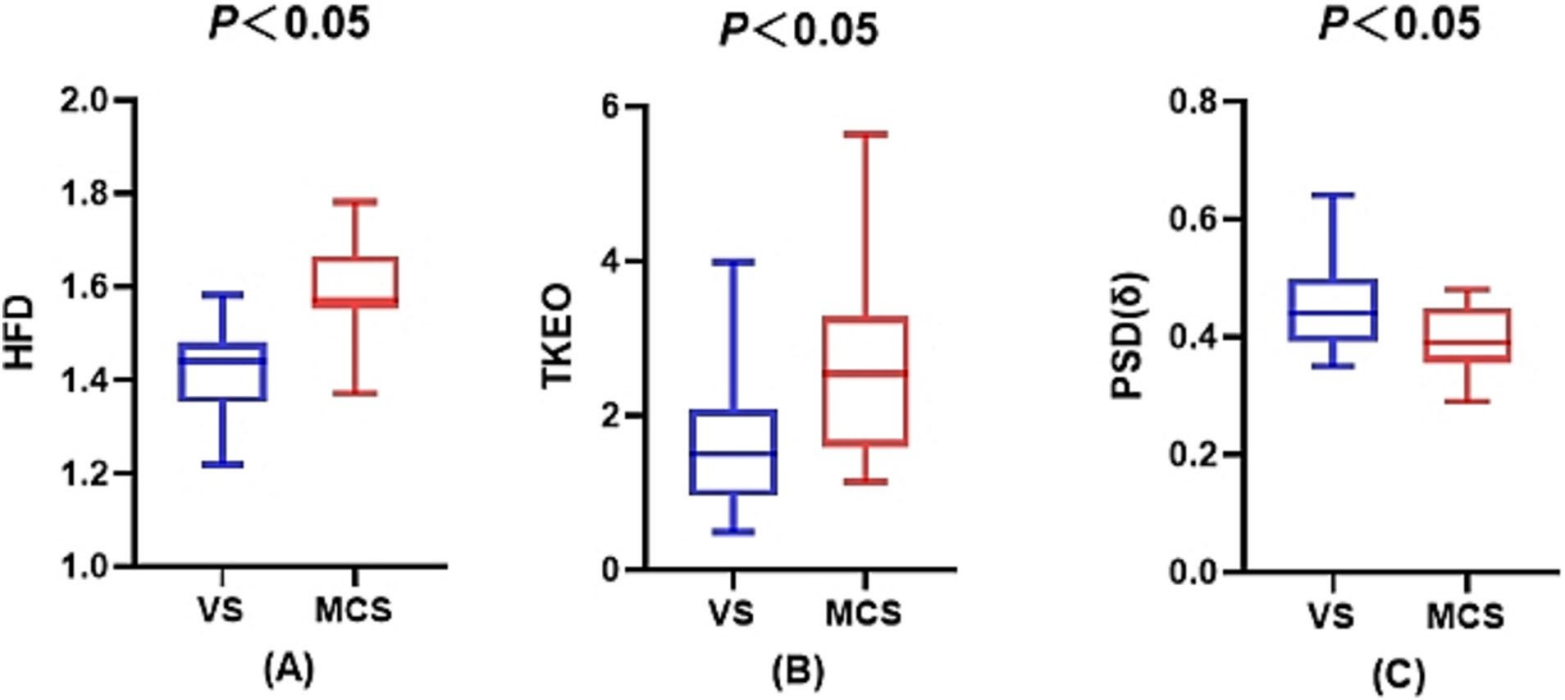
Sleep electroencephalogram differences between the VS/UWS and MCS groups. **(A)** The difference in the fractal dimension during wakefulness between the VS/UWS and MCS groups. **(B)** The difference in the Teager-Kaiser energy operator during the NREM2 phase between the VS/UWS and MCS groups. **(C)** The difference in the power spectral density of the *δ*-band during wakefulness between the VS/UWS and MCS groups.

The one-way ANOVA analysis revealed that the HFD in the MCS group exhibited a tendency to be higher than that in the VS/UWS group across all stages, with a statistically significant difference observed only during the waking stage (*p* < 0.001). Moreover, both groups displayed a gradual decrease in the HFD from the waking stage to NREM3, followed by an increase in the REM stage. Similarly, the TKEO in the MCS group showed a discernible tendency to be higher than that in the VS/UWS group across all stages, with a statistically significant difference observed only in the NREM2 stage (*p* = 0.009). Furthermore, the PSD (*δ*) in the MCS group demonstrated a pattern of being lower than that in the VS/UWS group across all stages, with statistical significance observed only during the waking stage (*p* = 0.017). For further details, please refer to Table 1 and Figure 2.

### 3.3 Spearman correlation analysis

**Figure 3:**
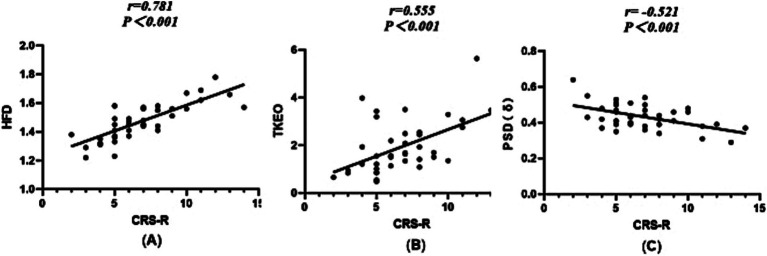
Scatterplot between the HFD, TKEO, PSD (δ), and CRS-R scores. **(A)** Dimension of the fractal during wakefulness. **(B)** The TKEO in the NREM2 phase. **(C)** The power spectral density of the δ-band during wakefulness.

The Spearman correlation analysis of the HFD, TKEO, PSD of *δ* waves, and CRS-R scores revealed significant relationships. The HFD showed a strong positive correlation with the CRS-R score (*r* = 0.781, *p* < 0.001). Similarly, the TKEO was positively correlated with the CRS-R score (*r* = 0.555, *p* < 0.001). In contrast, the PSD (δ) exhibited a negative correlation with the CRS-R score (*r* = −0.512, *p* < 0.001). Among these indicators, the HFD demonstrated the strongest correlation with the CRS-R score. Please refer to Figure 3 for further details.

### 3.4 Analysis of the receiver operating characteristic curve (ROC curve)

**Figure 4:**
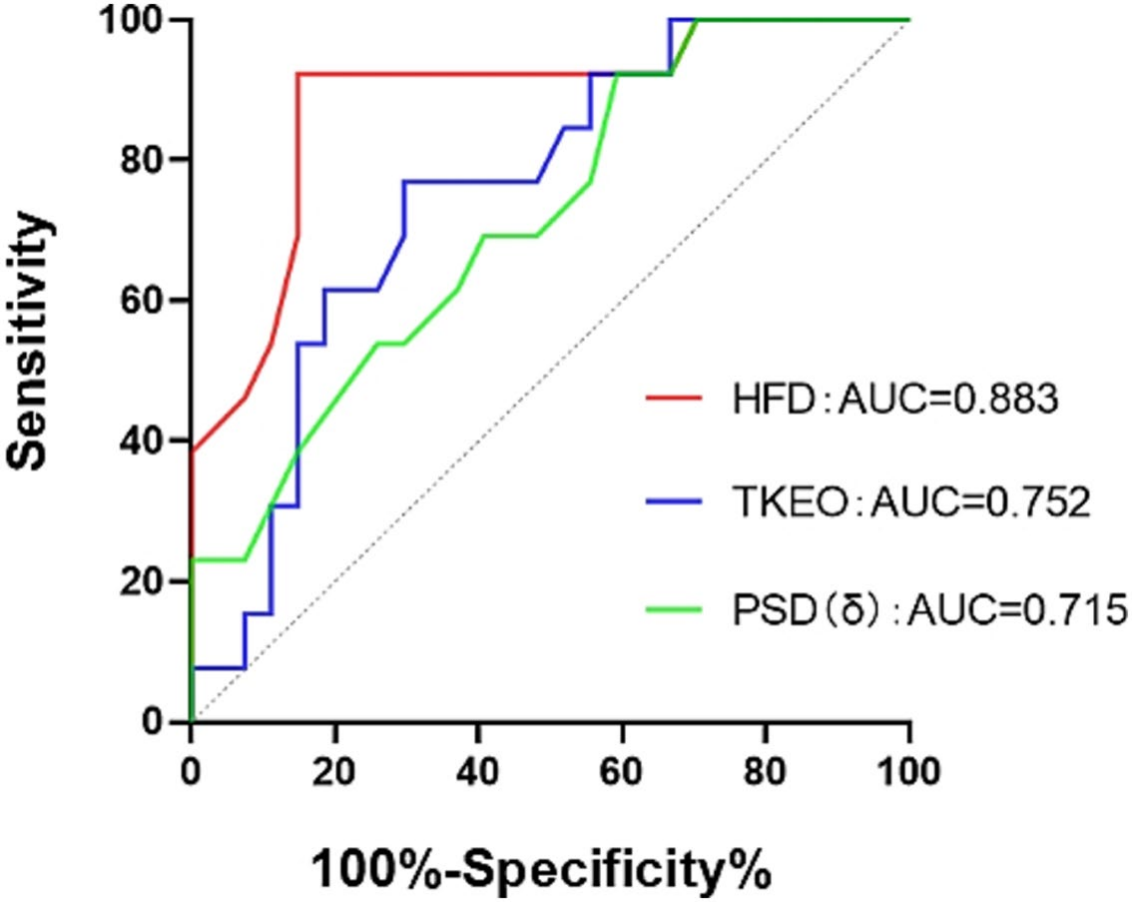
ROC curve. HFD, the fractal dimension during wakefulness. The TKEO, the Teager–Kaiser energy operator in the NREM2 phase. The PSD (δ), the power spectral density in the δ-band during wakefulness.

The ROC curve analysis indicated that the HFD exhibited the highest diagnostic accuracy at 88.3% (*p* < 0.001). The TKEO demonstrated diagnostic accuracy of 75.2% (*p* = 0.011), while the PSD of δ waves had diagnostic accuracy of 71.5% (*p* = 0.029). Please see Figure 4 for additional details.

## 4 Discussion

As the number of patients with pDOC continues to rise, an increasing number of studies have been conducted in recent years. Although the CRS-R score remains the gold standard for diagnosis, its misdiagnosis rate is notably high in clinical applications (Giacino et al., 2004). In contrast to neuroimaging techniques such as fMRI and PET, there is growing interest in electrophysiological techniques such as PSG and EEG. This interest stems from the advantages of electrophysiological testing as it is low-cost, non-invasive, and portable. Given that many patients with pDOC are bedridden and may have poor oxygen saturation, these techniques can be performed at the bedside, making them widely applicable in clinical practice (Comanducci et al., 2020; Porcaro et al., 2022). Moreover, unlike neuroimaging methods, electrophysiological techniques provide direct measurements of brain electrical activity, allowing for an intuitive reflection of changes in brain function. This makes them particularly valuable for diagnosing pDOC, a notion supported by the growing number of electrophysiological markers developed over the past decade (Comanducci et al., 2020; Porcaro et al., 2022). While PSG requires a longer detection time compared to EEG, it allows for the exploration of the complete sleep–wake cycle and the characteristics of EEG, revealing the close relationship between the conscious brain network and the sleep brain network (Duclos et al., 2017). Therefore, investigating sleep differences in pDOC using PSG is highly valuable. In this study, we analyzed the PSG data from 27 patients in the VS/UWS and 13 patients in the MCS, finding significant differences in the HFD and PSD (*δ*) during wakefulness, as well as in the TKEO during the NREM2 stage. The correlation analyses indicated that both HFD and TKEO were significantly positively correlated with the CRS-R scores, while the PSD (δ) exhibited a significant negative correlation with the CRS-R scores. Notably, the HFD showed the strongest correlation with the CRS-R scores (*r* = 0.781). The ROC curve analysis demonstrated significant diagnostic efficacy for the HFD, TKEO, and PSD (δ), with the HFD achieving the highest diagnostic accuracy of 88.3%.

HFD of EEG signals serves as an indicator of the complexity of EEG activity, with higher HFD values potentially reflecting more complex brain dynamics associated with elevated levels of consciousness. Currently, HFD is widely utilized in various fields, including neuroimaging analysis, bone studies, mammography, and ECG/EEG diagnostics (Maetani et al., 2023). Its versatility in EEG applications has contributed to significant advancements in understanding several disorders, such as epilepsy, sleep disorders, and Alzheimer’s disease (Gangadhar et al., 2003; Woyshville and Calabrese, 1994; Raghavendra et al., 2009; De Ruiz Miras et al., 2019). Woyshville et al. reported that patients with Alzheimer’s disease exhibited significantly lower HFD values compared to healthy participants (Woyshville and Calabrese, 1994). Similarly, Raghavendra et al. found a diffuse reduction in HFD values among patients with schizophrenia (Raghavendra et al., 2009). Ruiz et al. demonstrated that brain activity in the conscious state displayed a more complex structure and evolution compared to the unconscious state, noting that HFD values during sleep were generally lower than those during wakefulness (De Ruiz Miras et al., 2019). In our study, we observed that patients’ HFD values gradually decreased from wakefulness to deep sleep, with a tendency for the HFD to increase upon entering the REM phase, potentially correlating with dreaming during this stage (Boyce and Adamantidis, 2017). Furthermore, during the wakefulness period, the HFD values of the patients in the MCS were significantly higher compared to those in the VS/UWS. This finding suggests that brain activity in the MCS is more pronounced, indicating a higher level of consciousness. The correlation analysis further revealed that higher CRS-R scores corresponded with increased HFD values, reflecting greater complexity in brain activity. Although a similar trend was observed in the TKEO, it was not as pronounced as that seen with the HFD, aligning with previous studies.

The TKEO is a non-linear indicator that reflects the complexity of instantaneous energy in various signals. It has applications in engineering and materials science, where it is widely used to diagnose bearing faults (Shi et al., 2022) and to detect layered composite materials by analyzing instantaneous energy changes (Gałęzia and Orłowska-Gałęzia, 2021). In recent years, numerous studies have investigated the role of the TKEO in biological contexts. For instance, the TKEO has been shown to enhance the detection of electromyography onset (Li et al., 2007), identify focal EEG signals in patients with epilepsy (Chatterjee, 2019), quantify respiratory impacts on cardiac function by analyzing electrocardiogram changes (Imirzalioglu and Semiz, 2022), and detect spontaneous seizures in mice during basic experimental setups (Wei et al., 2021). However, no studies have yet explored the role of the TKEO in the sleep EEG of patients with pDOC. Our study found that during the NREM2 stage, the TKEO values were significantly higher in the patients in the MCS compared to those in the VS/UWS. This finding suggests that the EEG signals of patients in the MCS exhibit greater energy, further confirming that residual brain function in the MCS is relatively well-developed compared to the VS/UWS, with stronger functional connectivity among consciousness-related brain regions. The correlation analysis indicated a positive relationship between the TKEO and CRS-R scores, suggesting that the TKEO may serve as a sensitive indicator of consciousness level progression. Currently, there are limited studies on this topic, and further exploration of its clinical value is warranted.

Numerous previous studies on power spectra have identified significant differences between the power spectra of patients with pDOC and healthy controls, as well as between those in the VS/UWS and MCS (Stefan et al., 2018). Specifically, patients with pDOC demonstrated decreased *α*-band power and increased *δ*-band power, with these differences being more pronounced in individuals in the VS/UWS compared to those in the MCS (Franks, 2008). The PSD of δ waves is a commonly used linear metric that is closely associated with deep sleep stages, which are critical for assessing brain function, particularly in cases involving altered states of consciousness. The presence of stable and intense δ waves during anesthesia and deep sleep typically indicates unconsciousness (Franks, 2008). Our study reached a similar conclusion, finding that the PSD (δ) was significantly higher in the VS/UWS group than in the MCS group. However, the correlation with the CRS-R score was lower, and the diagnostic accuracy was also diminished, indicating weaker diagnostic value compared to the HFD and the TKEO.

The application of non-linear methods in EEG analysis has garnered increasing interest, recognizing that EEG production may not be solely explained by linear deterministic processes. As previously mentioned, Porcaro et al. found that HFD is more effective at detecting differences between the MCS and VS/UWS compared to traditional linear methods (Porcaro et al., 2022). In our study, both HFD and TKEO were identified as non-linear indicators, while the PSD of *δ* waves served as a linear indicator. We observed that the correlation between the HFD and TKEO and the CRS-R scores was significantly higher compared to the PSD (δ). In addition, the ROC curve analysis indicated that the diagnostic accuracy of both HFD and TKEO surpassed that of the PSD (δ), particularly for the HFD, which demonstrated a stronger correlation and higher diagnostic accuracy. This finding aligns with Porcaro et al.’s conclusion that the diagnostic efficacy of non-linear indicators may be superior to that of linear indicators, especially HFD, which shows promising diagnostic value in the assessment of pDOC.

In conclusion, our study identified three differential indicators in the PSG results of the patients in the MCS and VS/UWS. The correlation and ROC curve analyses demonstrated that all three differential indicators were correlated with the CRS-R scores and exhibited diagnostic value. Among these, the non-linear indicators (HFD and the TKEO) showed stronger correlations and higher diagnostic accuracy than the linear indicator, the PSD (*δ*), particularly HFD. This finding can help clinical practitioners distinguish between the VS/UWS and MCS, reduce the misdiagnosis rate, and provide an objective basis for clinical decision-making. However, we acknowledge that a primary limitation of the study is the relatively small sample size and the significant discrepancy in the number of participants between the two groups, which might have affected the outcomes. In addition, the 16-h testing period might not have encompassed the complete sleep–wake cycle of the patients compared to a 24-h PSG assessment. Therefore, further research is needed to validate our findings in a larger cohort and to increase the number of test leads and the duration of testing as much as possible. Furthermore, diagnosing pDOC is complex, and relying on single diagnostic methods is often insufficient; thus, we should explore as many multimodal diagnostic approaches as possible in the future.

## 5 Conclusion

The PSG results in the VS/UWS and MCS groups differed significantly, aiding in distinguishing between these groups and lowering the misdiagnosis rate. Notably, the HFD and PSD (δ) showed better diagnostic potential during wakefulness than during sleep, while the TKEO excelled in NREM2. In particular, the HFD during wakefulness exhibited a stronger correlation with the CRS-R scores, the highest diagnostic accuracy, and promise in diagnosing pDOC.

## Data Availability

The raw data supporting the conclusions of this article will be made available by the authors, without undue reservation.
