# Supplementary material for: Electroencephalographic differences between waking and sleeping periods in patients with prolonged disorders of consciousness at different levels of consciousness
Source: Front Hum Neurosci. 2025 Feb 17;19:1521355. doi: 10.3389/fnhum.2025.1521355 (PMC11872887; doi:10.3389/fnhum.2025.1521355)
Supplement: Supplementary file 1 [file Data_Sheet_1.DOCX]

Supplementary Material

##### 1.Power in Delta Frequency Bands

Power spectral density (PSD) is a measure that quantifies the power distribution of a signal across different frequency components^[1]^. In this study, PSD for each epoch was calculated using the Welch method. The delta band, encompassing brainwave frequencies typically ranging from 0.5 to 4 Hz, is strongly associated with deep sleep stages and is critical in assessing brain function, especially in conditions involving altered states of consciousness. Research has shown significant differences in power spectra between patients with disorders of consciousness (DOC) and healthy controls, as well as between those in unresponsive wakefulness syndrome (UWS) and minimally conscious state (MCS)^[2]^. Specifically, DOC patients exhibit reduced alpha-band power and increased delta-band power, with these differences being more pronounced in UWS patients compared to MCS patients. Moreover, the presence of stable and intense delta waves is indicative of unconsciousness, as observed in anesthesia and deep sleep^[3]^.

##### 2. Higuchi fractal dimension

The Higuchi fractal dimension (HFD) is a technique used to estimate the fractal dimension of discrete time series data, serving as a quantitative measure of the complexity or self-similarity inherent in a signal or geometric structure^[4]^. This method has been extensively applied in sleep research to analyze and characterize brain activity across different sleep stages, leveraging HFD’s ability to quantify the complexity of electroencephalogram (EEG) signals, which exhibit significant variations throughout the various stages of sleep^[5]^.Notably, the Higuchi method directly estimates the fractal dimension from the time series data without necessitating any prior transformation or embedding. For a time series $X(1),X(2),...,X(N)$ of length$N$, the HFD proceeds as follows：

$$X_{m}^{k}=\left\{X(m),X(m+k),X(m+2k),\ldots,X\left(m+\left[\frac{N-m}{k}\right]k\right)\right\}$$

where $m=1,2,3,...,k$. For each constructed time series$X_{m}^{k}$ , calculate the length$L_{m}(k)$:

$$L_{m}(k)=\frac{1}{k}\left(\sum_{i=1}^{\left[\frac{N-m}{k}\right]} |X(m+ik)-X(m+(i-1)k)|\right)\frac{(N-1)}{\left[\frac{N-m}{k}\right]k}$$

where $\left[\frac{N-m}{k}\right]k$is a normalization factor, and Compute the average length $L(k)$over all $m$:

$$L(k)=\frac{1}{k}\sum_{m=1}^{k} L_{m}(k)$$

The slope of the linear regression line fitted to these data points is utilized to estimate the fractal dimension $D$. Mathematically, $D$can be defined as follows:

$$D=-\frac{d\log(L(k))}{d\log(k)}$$

##### 3. DWT-TKEO

The discrete wavelet transform (DWT) is a powerful tool for analyzing non-stationary signals, as it provides a detailed representation of both time-domain and frequency-domain components, enabling precise analysis in the joint time-frequency domain ^[6]^. For a continuous signal$x(t)$,DWT can be defined as below:

where is wavelet function，$2^{\text{j}}$and$2^{\text{jk}}$are referred as the scaling and shifting parameters and the signal to be processed, respectively.

The Teager-Kaiser Energy Operator (TKEO) is a nonlinear energy-tracking operator capable of determining the instantaneous energy of nonstationary signals. Mathematically, TKEO can be defined as follows:

$$\psi(x[n])=x^{2}[n]-x[n-1]x[n+1]$$

This operator provides a measure of the energy and frequency content of the signal. For continuous-time signals, the TKEO is often expressed as：

where $\text{x(t)}$is the signal as a function of time $t$, is the first derivative of $x(t)$ with respect to time, is the second derivative of $x(t)$ with respect to time.

Initially, a 6-level discrete wavelet transform (DWT) is applied to decompose the signal into its frequency sub-bands. By applying the TKEO to each decomposed sub-band and utilizing equations (2) and (3), the instantaneous energy operator for each frequency sub-band of the EEG signal is obtained. Table 1 shows the statistical features (SF) utilized in the present work.

**Table 1 Frequency Sub-bands and the corresponding TKEO features**

| **Level of Decomposition** | **Range of Frequency（Hz）** | **TKEO mean**  **(Mean value)** | **TKEO standard deviation (Mean value)** |
| --- | --- | --- | --- |
| D1 | 50-25 | teager_kaiser_energy12 | teager_kaiser_energy13 |
| D2 | 25-12.5 | teager_kaiser_energy10 | teager_kaiser_energy11 |
| D3 | 12.5-6.25 | teager_kaiser_energy8 | teager_kaiser_energy9 |
| D4 | 6.25-3.125 | teager_kaiser_energy6 | teager_kaiser_energy7 |
| D5 | 3.125-1.56 | teager_kaiser_energy4 | teager_kaiser_energy5 |
| D6 | 1.56-0.78 | teager_kaiser_energy2 | teager_kaiser_energy3 |
| A6 | 0.78-0 | teager_kaiser_energy0 | teager_kaiser_energy1 |

## **Reference**

[1] L. Bonfiglio *et al.*, “Cortical source of blink-related delta oscillations and their correlation with levels of consciousness: Delta BROs and Consciousness,” *Hum. Brain Mapp.*, vol. 34, no. 9, pp. 2178–2189, Sep. 2013, doi: 10.1002/hbm.22056.

[2] S. Stefan *et al.*, “Consciousness Indexing and Outcome Prediction with Resting-State EEG in Severe Disorders of Consciousness,” *Brain Topogr.*, vol. 31, no. 5, pp. 848–862, Sep. 2018, doi: 10.1007/s10548-018-0643-x.

[3] N. P. Franks, “General anaesthesia: from molecular targets to neuronal pathways of sleep and arousal,” *Nat. Rev. Neurosci.*, vol. 9, no. 5, pp. 370–386, May 2008, doi: 10.1038/nrn2372.

[4] R. A. Ganesan and R. Jain, “Binary State Prediction of Sleep or Wakefulness Using EEG and EOG Features,” in *2020 IEEE 17th India Council International Conference (INDICON)*, in 分形维数. Dec. 2020, pp. 1–7. doi: 10.1109/INDICON49873.2020.9342272.

[5] W. Klonowski, E. Olejarczyk, and R. Stepien, “Sleep-EEG Analysis Using Higuchi’s Fractal Dimension”.

[6] M. Li, W. Chen, and T. Zhang, “Classification of epilepsy EEG signals using DWT-based envelope analysis and neural network ensemble,” *Biomed. Signal Process. Control*, vol. 31, pp. 357–365, Jan. 2017, doi: 10.1016/j.bspc.2016.09.008.
